# Supervised Natural Language Processing Classification of Violent Death Narratives: Development and Assessment of a Compact Large Language Model

**DOI:** 10.2196/68212

**Published:** 2025-06-19

**Authors:** Susan T Parker

**Affiliations:** 1 Feinberg School of Medicine, Northwestern University, 750 N Lakeshore, Chicago, IL, 60611, United States, 1 2487613116

**Keywords:** natural language processing, NLP, violence, informatics, text classification, simulation, violent death, narrative, large language model, LLM, injury prevention, violent injury, coroner reports, police report

## Abstract

**Background:**

The recent availability of law enforcement and coroner or medical examiner reports for nearly every violent death in the United States expands the potential for natural language processing (NLP) research into violence.

**Objective:**

The objective of this work is to assess applications of supervised NLP to unstructured data in the National Violent Death Reporting System to predict circumstances and types of violent death.

**Methods:**

This analysis applied distilBERT, a compact large language model (LLM) with fewer parameters relative to full-scale LLMs, to unstructured narrative data to simulate the impacts of preprocessing, volume, and composition of training data on model performance, evaluated by *F*_1_-scores, precision, recall, and the false negative rate. Model performance was evaluated for bias by race, ethnicity, and sex by comparing *F*_1_-scores across subgroups.

**Results:**

A minimum training set of 1500 cases was necessary to achieve an *F*_1_-score of 0.6 and a false negative rate of 0.01-0.05 with a compact LLM. Replacement of domain-specific jargon improved model performance, while oversampling positive class cases to address class imbalance did not substantially improve *F*_1_-scores. Between racial and ethnic groups, *F*_1_-score disparities ranged from 0.2 to 0.25, and between male and female decedents, differences ranged from 0.12 to 0.2.

**Conclusions:**

Compact LLMs with sufficient training data can be applied to supervised NLP tasks with a class imbalance in the National Violent Death Reporting System. Simulations of supervised text classification across the model-fitting process of preprocessing and training compact LLM-informed NLP applications to unstructured death narrative data.

## Introduction

Violent injuries are among the leading causes of death in the United States for individuals younger than the age of 44 years and are leading causes for young people aged 10‐34 years [1]. The most comprehensive and detailed source of data on violent deaths in the United States is the National Violent Death Reporting System (NVDRS), aggregating information from death certificates, coroner or medical examiner reports, and law enforcement (LE) reports to characterize violent deaths [2]. Researchers have used structured data from NVDRS extensively to characterize the epidemiology of violent deaths including homicides [3-6], suicides [7-10], and those that result from legal intervention (police shootings) [11].

NVDRS has been widely used for its structured data, which captures information such as decedent characteristics, weapons, circumstances, and suspect information [12], and increasing attention has been given to the vast amounts of unstructured text data embedded within the narrative reports. Narratives provide rich details about the incident not necessarily captured in structured variables, such as nuanced descriptions of precipitating events and other contextual factors that are difficult to quantify.

A range of approaches have been applied to the use of NVDRS narratives in research on violent deaths. According to a recent review on the research use of textual NVDRS narratives over the past 2 decades, most studies used manual review or keyword searches of narratives [10,13,14], while 5% used machine learning tools designed to analyze unstructured text, known as natural language processing (NLP) [15]. Applications of NLP have included supervised learning tasks, such as classification, as well as unsupervised tasks, such as topic modeling. For instance, supervised NLP has been used to classify suicide related to driving cessation [16] and assisted living facilities [17], examine suicide intent classification [18] and intimate partner homicide [19], and predict drug overdose deaths [20]. Latent class analysis has been used to reveal salient topics unrepresented in abstractor classification [21,22] and themes in youth suicide [23]. Most recently, researchers have used NLP to classify social determinants of health in suicide narratives [24] and inconsistencies, biases, and missing data in the narratives themselves [25-27].

Continued application of NLP to NVDRS is particularly important because the volume of NVDRS data will substantially increase over time. NVDRS has gathered data on over 500,000 deaths since 2003 and will grow by approximately 100,000 records annually moving forward as additional states and counties participate, underlining the importance of efficiently investigating research questions using NVDRS narratives and NLP methods.

Although large language models (LLMs) have generally performed better than other NLP approaches to narrative data in medical informatics domains, fewer applications of LLMs to NVDRS exist [24,28]. Applications of NLP to a related text narrative type, clinical notes from medical providers, have identified patient self-harm [29-34] and violence-related [35-38] outcomes often using LLMs or deep learning approaches. In part, researchers and practitioners may face particular challenges applying LLMs to NVDRS. One important challenge is that many outcomes of interest are likely to be infrequent or rare events that can present classification challenges due to sparse information about the outcome [39-41]. Further, NVDRS narratives are composed of police and coroner reports, which contain domain-specific language or jargon, such as the use of *ICD* (*International Classification of Disease*) codes. NVDRS data restrictions on sensitive data do not permit narratives to be stored in the cloud, thus limiting access to computing resources that are often used to train or fine-tune LLMs. Fourth, researchers documented racial disparities in narratives alongside gendered text differences in NVDRS [22,26,27]. Narratives involving victims from marginalized populations tend to be significantly shorter in length and are more likely to be missing altogether. These differences in data quality may result in models that generate predictions with similar patterns of subgroup bias.

To address these challenges, this paper conducts simulations of supervised text classification that span the machine learning pipeline, from data preprocessing and model training to the evaluation of predictions for potential racial or gender bias. Existing coded variables that record the type (eg, police shooting and drive-by shooting) or circumstances (number of nonfatal shooting victims and location of victim injuries) of the violent death are used as target outcomes used in simulations. Target outcomes with class imbalance were selected, as this setting is likely of most use to NVDRS applications, and models were fit using a compact LLM to reflect settings where computing resources are limited. By conducting simulations, this analysis aims to inform future applications of supervised classification using LLMs to NVDRS by establishing concrete benchmarks for understanding training data quantity, preprocessing needs, and to what extent NLP results in predictions reflecting existing racial or gender bias in narratives.

## Methods

### Data

This analysis used violent death records from NVDRS data from 2015 to 2020. The NVDRS gathers information about violent deaths including homicides, suicides, and deaths caused by LE. NVDRS combines data from death certificates, coroner or medical reports, and LE reports, providing context about violent deaths including information about mental health conditions, toxicology results, and other circumstances in addition to details about victim characteristics. Trained medical abstractors code information from reports about violent deaths into the over 600 variables that comprise the NVDRS surveillance system [12].

To obtain labeled outcomes for use as target outcomes in simulations, this analysis constructed measures from existing coded NVDRS variables that abstractors label. Because a substantial proportion of coded NVDRS fields group together case outcomes that are negative with those that are not known, this analysis instead relied on multinomial fields or combined separate NVDRS coded variables to obtain target outcomes for simulations. For instance, for case outcomes such as mental health crisis or drug involvement, outcomes are coded as “Yes” or as “No, not available, unknown,” which would not constitute a labeled outcome.

These constructed outcomes include 4 binary outcomes likely to be recorded accurately when known. The first outcome is whether or not a homicide is a legal intervention homicide, meaning the shooter was a LE officer. Literature suggests that these homicides are well-recorded in NVDRS and less subject to noisy labeling or measurement error [11]. The second outcome is whether or not a homicide is classified as a drive-by shooting. The third outcome is whether a homicide occurred at home or not, and the fourth outcome is whether or not additional victims were nonfatally shot in the course of a homicide event. We constrain the sample to where the weapon type is listed as a firearm, and the abstractor manner of death is a homicide. Taken together, these outcomes represent a range of language complexity and frequency less subject to label noise by constructing outcomes.

### Ethical Considerations

The Northeastern University institutional review board deemed that this research did not require review, as it did not involve human participants.

### Statistical Analysis

This analysis compared model performance across 4 configurations of training data and text composition using a compact LLM. The configurations examined included preprocessing of text data as well as the amount and composition of the training data. Specifically, the analysis first varied the amount of training data that the model was fitted on to inform how much randomly sampled training data must be annotated to train an LLM to predict NVDRS outcomes. Second, because positive class cases were often infrequent, the analysis simulated the oversampling of positive class cases in training data. Specifically, oversampling included a larger proportion of additional positive class cases, holding the negative class cases constant, to inform what composition of training data was most effective to include as training data.

This analysis additionally simulated different preprocessing techniques for unstructured text data. NVDRS text may be domain-specific, as it comprises police and coroner reports, which use both jargon and abbreviation. To simulate the impacts of clarifying common abbreviations, this analysis replaced NVDRS abbreviations with unabbreviated text. For example, often when NVDRS abstractors referred to victims and suspects in the report narratives, the abbreviations “v” for victim and “s” for suspect appeared rather than the full word. Abbreviations referring to victims, suspects, police, and gunshot wounds were replaced (Table S1 in Multimedia Appendix 1).

Finally, the analysis simulated omitting coroner report text from the training data. Coroner reports may contain extraneous text such as toxicology reports that may be noisy in the context of prediction focused on criminal justice outcomes. Further, compact LLMs have limited token lengths, which constrain the number of words in an input narrative, and the combination of coroner and homicide reports can exceed the token length in some LLM applications. Because our outcomes are LE-focused, the analysis simulated the omission of potentially extraneous narrative information.

The analysis began by preprocessing the coroner and police narrative by removing special characters including numbers, punctuation, and capitalization as is standard. Police and coroner report narratives were combined into a single field in order to use the information available in both narratives (with the exception of the LE narrative–only simulation).

Next, the analysis turned to creating simulated data. First, a test set on which the model outputs were to be evaluated was randomly selected. The test set consisted of a random sample of 30% of each outcome’s records, which was then held out from any selection into the training data.

To vary the amounts of training data, the analysis used different training data record counts, each with a different amount of training data. These splits ranged from a minimum of 100 cases, increasing in increments to 200, 500, 1000, 1500, and up to 2000 cases. Each split was randomly sampled from the full dataset specified for each outcome so that each training split maintained a proportion of positive and negative cases that approximates the true proportion. The prior sample was included in the next iteration to isolate the impact of adding additional training data, not adding different training data. For instance, to obtain 500 cases, first, the prior 200 cases were preserved, and an additional 300 were sampled to comprise 500 cases.

To simulate the impacts of language replacement and LE-only text, the analysis followed the procedure process outlined earlier to randomly select training data in the same 100, 200, 500, 1000, 1500, and 2000 increments.

In the second configuration of training data, the composition of positive class cases was altered from the true proportion in the training data. Instead of randomly sampling cases, the proportion of positive class cases was increased in the training data by adding additional positive class cases to the negative class cases. The positive class cases were incrementally increased until they comprise 10%, 20%, 30%, 40%, and up to 50% of the training data starting from a baseline of 1000 cases, as lower amounts of training data were not performant in this application. For instance, to obtain training data composed of 10% positive class cases for legal intervention homicide, the process started with randomly sampled training data with 1000 records, of which 54 were legal intervention homicides and 940 were not. To the 940 negative class cases, 59 additional positive class cases were added so that the total number of positive class cases was 113 (54+59), and the total was 1059 cases, of which approximately 10% (113/1059) were legal intervention homicides.

For each of the configurations described earlier, distilBERT, an LLM with fewer parameters but comparable accuracy to large-scale LLMs, was used [41]. Compact LLMs better allow for simulations because of fewer computational needs and because NVDRS data restrictions do not permit cloud storage and computing. The distilBERT models were fine-tuned on training data to select model parameters. Parameters were selected in initial fine-turning using 2 outcomes (legal intervention and drive-by). Because model parameters in each fine-tuned model were identical, these parameters were applied to each training data configuration (Table S2 in Multimedia Appendix 1). Because our target outcomes are imbalanced, we add a weighted trainer to account for class imbalance. Configurations are summarized in Table S3 in Multimedia Appendix 1.

Classification performance was measured using learning curves, which plot performance metrics relative to differing splits of labeled training data to evaluate classifier model performance. Binary classification model metrics including precision and recall in addition to metrics considered useful for imbalanced class problems, including an *F*_1_-score, were used. Finally, to analyze classification performance by subgroup, learning curves were created for sex, race, and ethnicity subgroups.

## Results

Classification outcomes differed by the proportion of positive to negative cases in each outcome (Table 1). The most rare positive class outcome was a police shooting (n=4489, 5.9%) followed by drive-by shootings (n=6575, 9.2%) and shootings where additional individuals were nonfatally shot (n=8052, 15.2%) in the course of the homicide. The most prevalent outcome was whether an individual is shot in their home (n=16,850, 24.8%) relative to another location outside the home. Victims of homicide in the sample tended to be male (n=4319-11,321; 67.2-96.2%), Black or African American (n=44,546-43,357, 58.5%‐60.5%), and young, with the most frequent age range between 25 and 34 years (Table 1). Intimate partner violence characterizes over a tenth of homicides overall but within cases where an individual is injured at home, intimate partner violence (n=17,226, 26.5%) occurred in over a quarter of cases. Legal intervention homicides were most likely associated with mental health problems and alcohol use.

Circumstances were known for almost all cases of legal intervention and drive-by shootings, but less information was known about the circumstances of homicide where additional individuals were shot or when they were injured at home (Table 2). Circumstances were known in 71% (n=30,774) of homicides of Black decedents in contrast to 83.7% (n=8698) among Hispanic and non-Hispanic White decedents. The median number of words in a narrative for a LE narrative ranged from 80‐83, whereas coroner and medical examiner narratives ranged from 88 to 91 words in length. Legal intervention homicides had the most lengthy narratives (115 for LE and 120 for coroner and medical examiner). Narrative length differed by race and sex. Among LE narratives, the median length for Black decedents was 98 words but 132 for non-Hispanic White decedents. Narrative length differed among male and female decedents. Female decedents had longer narratives for each homicide outcome. Female decedents shot at home had a median narrative length of 124 words in contrast to male decedents shot at home with a length of 92 words.

**Table 1.** Sample descriptive statistics, characteristics by outcome.

Variable	Drive-by			Legal intervention			Number nonfatally shot			Individual injured at home		
	Overall (n=71,708), n (%)	Negative case (n=65,133), n (%)	Positive case (n=6575), n (%)	Overall (n=76,197), n (%)	Negative case (n=71,708), n (%)	Positive case (n=4489), n (%)	Overall (n=53,024), n (%)	Negative case (n=44,972), n (%)	Positive case (n=8052), n (%)	Overall (n=68,016), n (%)	Negative case (n=51,166), n (%)	Positive case (n=16,850), n (%)
Sex												
Female	11,255 (15.7)	10,587 (16.3)	668 (10.2)	11,425 (15)	11,255 (15.7)	170 (3.8)	8378 (15.8)	7043 (15.7)	1335 (16.6)	10,744 (15.8)	5215 (10.2)	5529 (32.8)
Male	60,447 (84.3)	54,540 (83.7)	5907 (89.8)	64,766 (85)	60,447 (84.3)	4319 (96.2)	44,640 (84.2)	37,923 (84.3)	6717 (83.4)	57,272 (84.2)	45,951 (89.8)	11,321 (67.2)
Race or ethnicity												
American Indian or Alaska Native, non-Hispanic	753 (1.1)	712 (1.1)	41 (0.6)	897 (1.2)	753 (1.1)	144 (3.2)	612 (1.2)	544 (1.2)	68 (0.8)	717 (1.1)	510 (1)	207 (1.2)
Asian or Pacific Islander, non-Hispanic	806 (1.1)	768 (1.2)	38 (0.6)	868 (1.1)	806 (1.1)	62 (1.4)	601 (1.1)	513 (1.1)	88 (1.1)	773 (1.1)	526 (1)	247 (1.5)
Black or African American, non-Hispanic	43,357 (60.5)	38,976 (59.8)	4381 (66.6)	44,546 (58.5)	43,357 (60.5)	1189 (26.5)	31,293 (59)	25,925 (57.6)	5368 (66.7)	41,109 (60.4)	33,639 (65.7)	7470 (44.3)
Hispanic	10,388 (14.5)	8745 (13.4)	1643 (25)	11,182 (14.7)	10,388 (14.5)	794 (17.7)	8116 (15.3)	6898 (15.3)	1218 (15.1)	9977 (14.7)	8098 (15.8)	1879 (11.2)
White, non-Hispanic	15,457 (21.6)	15,049 (23.1)	408 (6.2)	17,654 (23.2)	15,457 (21.6)	2197 (48.9)	11,687 (22)	10,476 (23.3)	1211 (15)	14,589 (21.4)	7772 (15.2)	6817 (40.5)
Age bins (years)												
15-24	19,403 (27.1)	17,012 (26.1)	2391 (36.4)	20,052 (26.3)	19,403 (27.1)	649 (14.5)	14,421 (27.2)	11,642 (25.9)	2779 (34.5)	18,454 (27.1)	15,811 (30.9)	2643 (15.7)
25-34	21,065 (29.4)	18,981 (29.1)	2084 (31.7)	22,334 (29.3)	21,065 (29.4)	1269 (28.3)	15,523 (29.3)	13,106 (29.1)	2417 (30)	20,011 (29.4)	16,324 (31.9)	3687 (21.9)
35-44	11,759 (16.4)	10,907 (16.7)	852 (13)	12,758 (16.7)	11,759 (16.4)	999 (22.3)	8595 (16.2)	7510 (16.7)	1085 (13.5)	11,136 (16.4)	8128 (15.9)	3008 (17.9)
45-54	6446 (9)	6088 (9.3)	358 (5.4)	7074 (9.3)	6446 (9)	628 (14)	4811 (9.1)	4303 (9.6)	508 (6.3)	6097 (9)	3716 (7.3)	2381 (14.1)
55-64	3545 (4.9)	3378 (5.2)	167 (2.5)	3897 (5.1)	3545 (4.9)	352 (7.8)	2591 (4.9)	2333 (5.2)	258 (3.2)	3330 (4.9)	1655 (3.2)	1675 (9.9)
65+	2452 (3.4)	2364 (3.6)	88 (1.3)	2601 (3.4)	2452 (3.4)	149 (3.3)	1806 (3.4)	1612 (3.6)	194 (2.4)	2309 (3.4)	687 (1.3)	1622 (9.6)
Unknown	6327 (8.8)	5771 (8.9)	556 (8.5)	6766 (8.9)	6327 (8.8)	439 (9.8)	4773 (9)	4085 (9.1)	688 (8.5)	5999 (8.8)	4486 (8.8)	1513 (9)
Intimate partner violence												
No, not available, unknown	64,071 (89.3)	57,642 (88.5)	6429 (97.8)	68,095 (89.4)	64,071 (89.3)	4024 (89.6)	47,096 (88.8)	39,667 (88.2)	7429 (92.3)	60,535 (89)	48,145 (94.1)	12,390 (73.5)
Yes	7637 (10.7)	7491 (11.5)	146 (2.2)	8102 (10.6)	7637 (10.7)	465 (10.4)	5928 (11.2)	5305 (11.8)	623 (7.7)	7481 (11)	3021 (5.9)	4460 (26.5)
Mental health problem												
No, not available, unknown	69,794 (97.3)	63,323 (97.2)	6471 (98.4)	73,436 (96.4)	69,794 (97.3)	3642 (81.1)	51,544 (97.2)	43,633 (97)	7911 (98.2)	66,149 (97.3)	50,059 (97.8)	16,090 (95.5)
Yes	1914 (2.7)	1810 (2.8)	104 (1.6)	2761 (3.6)	1914 (2.7)	847 (18.9)	1480 (2.8)	1339 (3)	141 (1.8)	1867 (2.7)	1107 (2.2)	760 (4.5)
Alcohol result												
Not present	29,990 (41.8)	26,479 (40.7)	3511 (53.4)	31,916 (41.9)	29,990 (41.8)	1926 (42.9)	23,045 (43.5)	19,419 (43.2)	3626 (45)	29,385 (43.2)	22,307 (43.6)	7078 (42)
Present	16,373 (22.8)	14,834 (22.8)	1539 (23.4)	17,732 (23.3)	16,373 (22.8)	1359 (30.3)	12,812 (24.2)	10,658 (23.7)	2154 (26.8)	16,044 (23.6)	12,691 (24.8)	3353 (19.9)
Unknown or not applicable	25,345 (35.3)	23,820 (36.6)	1525 (23.2)	26,549 (34.8)	25,345 (35.3)	1204 (26.8)	17,167 (32.4)	14,895 (33.1)	2272 (28.2)	22,587 (33.2)	16,168 (31.6)	6419 (38.1)
Argument												
No, not available, unknown	54,036 (75.4)	48,348 (74.2)	5688 (86.5)	57,841 (75.9)	54,036 (75.4)	3805 (84.8)	39,380 (74.3)	33,594 (74.7)	5786 (71.9)	50,790 (74.7)	38,887 (76)	11,903 (70.6)
Yes	17,672 (24.6)	16,785 (25.8)	887 (13.5)	18,356 (24.1)	17,672 (24.6)	684 (15.2)	13,644 (25.7)	11,378 (25.3)	2266 (28.1)	17,226 (25.3)	12,279 (24)	4947 (29.4)

**Table 2.** Narrative descriptive statistics, characteristics by outcome.

Variable	Drive-by			Legal intervention			Number nonfatally shot			Individual injured at home		
	Total cases	Negative class cases	Positive class cases	Total cases	Negative class cases	Positive class cases	Total cases	Negative class cases	Positive class cases	Total cases	Negative class cases	Positive class cases
Panel A: overall												
Circumstances known, n (%)	54,354 (75.8)	47,779 (73.4)	6575 (100)	58,745 (77.1)	54,354 (75.8)	4391 (97.8)	41,794 (78.8)	34,852 (77.5)	6942 (86.2)	52,852 (77.7)	38,879 (76)	13,973 (82.9)
Words law enforcement narrative, median (IQR)	80.0 (37.0-137.0)	78.0 (35.0-136.0)	93.0 (53.0-148.0)	81.0 (37.0-141.0)	80.0 (37.0-137.0)	115.0 (44.0-206.0)	83.0 (41.0-141.0)	78.0 (39.0-134.0)	113.0 (65.0-173.0)	83.0 (40.0-141.0)	80.0 (39.0-132.0)	94.0 (45.0-168.0)
Words CME^a^ narrative, median (IQR)	89.0 (55.0-138.0)	88.0 (54.0-137.0)	100.0 (64.0-147.0)	90.0 (56.0-141.0)	89.0 (55.0-138.0)	120.0 (78.0-182.0)	88.0 (56.0-137.0)	84.0 (53.0-132.0)	108.0 (72.0-163.0)	91.0 (58.0-140.0)	89.0 (56.0-134.0)	100.0 (62.0-161.0)
Panel B: Black												
Circumstances known, n (%)	30,774 (71)	26,393 (67.7)	4381 (100)	31,930 (71.7)	30,774 (71)	1156 (97.2)	23,041 (73.6)	18,562 (71.6)	4479 (83.4)	29,875 (72.7)	24,054 (71.5)	5821 (77.9)
Words law enforcement narrative, median (IQR)	77.0 (38.0-125.0)	74.0 (36.0-121.0)	98.0 (59.0-152.0)	77.0 (38.0-126.0)	77.0 (38.0-125.0)	98.0 (41.0-169.0)	80.0 (42.0-126.0)	74.0 (39.0-118.0)	109.0 (66.5-160.0)	80.0 (41.0-127.0)	79.0 (41.0-125.0)	82.0 (43.0-137.0)
Words CME narrative, median (IQR)	85.0 (55.0-126.0)	83.0 (53.0-123.0)	105.0 (72.0-151.0)	86.0 (55.0-127.0)	85.0 (55.0-126.0)	105.0 (73.0-150.0)	84.0 (56.0-125.0)	80.0 (53.0-119.0)	106.0 (72.0-155.0)	87.0 (57.0-128.0)	87.0 (57.0-126.0)	89.0 (58.0-137.0)
Panel C: Hispanic												
Circumstances known, n (%)	8698 (83.7)	7055 (80.7)	1643 (100)	9487 (84.8)	8698 (83.7)	789 (99.4)	7107 (87.6)	5980 (86.7)	1127 (92.5)	8521 (85.4)	6906 (85.3)	1615 (85.9)
Words law enforcement narrative, median (IQR)	67.0 (29.0-136.0)	66.0 (24.0-139.0)	72.0 ( 40.0-129.0)	69.0 (28.0-144.0)	67.0 (29.0-136.0)	132.0 (13.0-248.0)	69.0 (34.0-142.0)	65.0 (32.0-132.0)	106.0 (52.0-186.0)	69.0 (31.0-139.0)	66.0 (31.0-130.0)	89.0 (32.0-186.0)
Words CME narrative, median (IQR)	87.0 (45.0-146.0)	88.0 (48.0-149.0)	79.0 (31.0-134.0)	90.5 (47.0-151.0)	87.0 (45.0-146.0)	139.0 (90.0-206.0)	79.0 (41.0-141.0)	75.0 (39.0-134.0)	106.0 (60.0-170.0)	88.0 (47.0-147.0)	83.0 (44.0-141.0)	111.0 (66.0-180.0)
Panel D: White												
Circumstances known, n (%)	12,860 (83.2)	12,452 (82.7)	408 (100)	15,003 (85)	12,860 (83.2)	2143 (97.5)	10,052 (86)	8952 (85.5)	1100 (90.8)	12,496 (85.7)	6537 (84.1)	5959 (87.4)
Words law enforcement narrative, median (IQR)	99.0 (42.0-182.0)	99.0 (41.0-181.0)	112.0 (64.5-186.5)	102.0 (43.0-186.0)	99.0 (42.0-182.0)	119.0 (53.0-212.0)	105.0 (48.0-186.0)	101.0 (47.0-181.0)	134.0 (76.0-232.0)	104.0 (47.0-187.0)	97.0 (43.0-172.0)	114.0 (52.0-205.0)
Words CME narrative, median (IQR)	103.0 (61.0-165.0)	102.0 (61.0-165.0)	107.0 (71.5-149.5)	105.0 (63.0-167.0)	103.0 (61.0-165.0)	122.0 (77.0-184.0)	104.0 (64.0-166.0)	103.0 (63.0-163.0)	118.0 (74.0-191.0)	106.0 (65.0-168.0)	101.0 (63.0-156.0)	112.0 (68.0-183.0)
Panel E: female												
Circumstances known, n (%)	9404 (83.6)	8736 (82.5)	668 (100)	9567 (83.7)	9404 (83.6)	163 (95.9)	7242 (86.4)	6065 (86.1)	1177 (88.2)	9169 (85.3)	4271 (81.9)	4898 (88.6)
Words law enforcement narrative, median (IQR)	104.0 (48.0-184.0)	104.0 (46.0-186.0)	109.0 (60.0-158.5)	104.0 (48.0-184.0)	104.0 (48.0-184.0)	121.5 (48.0-193.0)	109.0 (54.0-189.0)	106.0 (51.0-186.0)	125.0 (69.0-204.0)	107.0 (51.5-187.0)	97.0 (47.0-165.0)	118.0 (57.0-207.0)
Words CME narrative, median (IQR)	111.0 (68.0-181.0)	112.0 (68.0-183.0)	105.0 (67.0-152.5)	111.0 (68.0-181.0)	111.0 (68.0-181.0)	134.0 (90.0-198.0)	112.5 (71.0-181.0)	111.0 (69.0-181.0)	122.0 (80.0-188.0)	113.0 (71.0-184.0)	105.0 (66.0-165.0)	124.0 (75.0-198.0)
Panel F: male												
Circumstances known, n (%)	44,950 (74.4)	39,043 (71.6)	5907 (100)	49,178 (75.9)	44,950 (74.4)	4228 (97.9)	34,552 (77.4)	28,787 (75.9)	5765 (85.8)	43,683 (76.3)	34,608 (75.3)	9075 (80.2)
Words law enforcement narrative, median (IQR)	76.0 (35.0-130.0)	74.0 (34.0-128.0)	92.0 (52.0-147.0)	78.0 (36.0-134.0)	76.0 (35.0-130.0)	114.0 (44.0-207.0)	79.0 (40.0-133.0)	74.0 (37.0-125.0)	110.0 (64.0-168.0)	79.0 (39.0-133.0)	78.0 (39.0-129.0)	85.0 (41.0-150.0)
Words CME narrative, median (IQR)	86.0 (53.0-131.0)	84.0 (53.0-129.0)	100.0 (64.0-146.0)	87.0 (55.0-135.0)	86.0 (53.0-131.0)	120.0 (78.0-181.0)	84.0 (54.0-129.0)	81.0 (52.0-124.0)	106.0 (70.0-158.0)	88.0 (56.0-133.0)	87.0 (55.0-131.0)	92.0 ( 58.0-145.0)

a CME: coroner and medical examiner.

**Figure 1.**
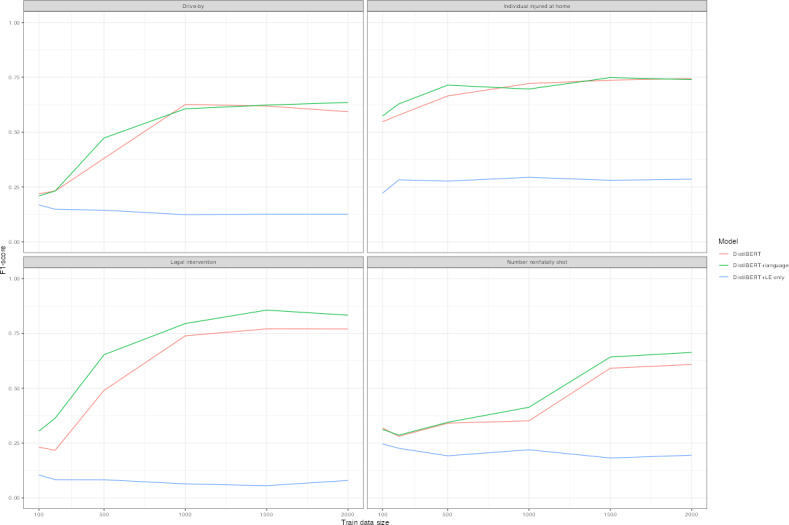
Learning curve by outcome, model type*—F*_1_-score. *F*_1_-scores are plotted for distilBERT models, distilBERT models with language replacement, and models that do not use LE narratives. Training data randomly sampled and corresponding to amounts of 100, 200, 500, 1000, 1500, and 2000 randomly sampled training datasets are plotted according to each model performance metric. Test sets reporting results are identical across models for each outcome variable. LE: law enforcement.

**Figure 2.**
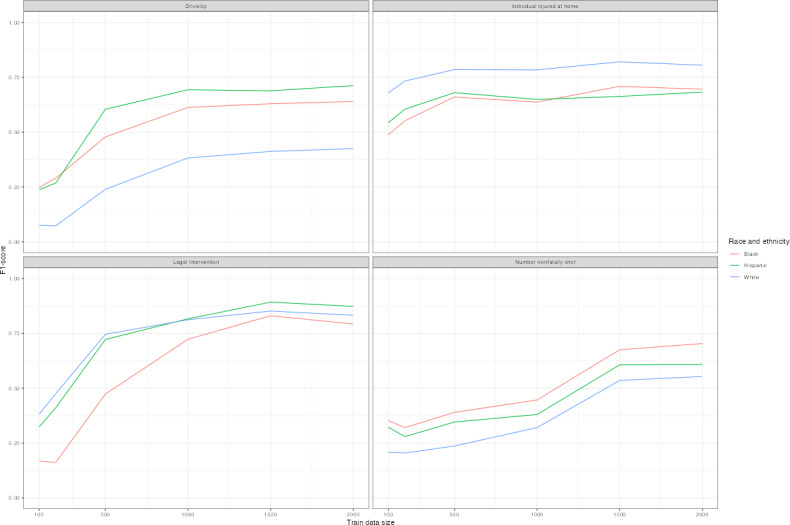
Learning curve by outcome, model type—precision. Precision scores are plotted for distilBERT models, distilBERT models with language replacement, and models that do not use LE narratives. Training data randomly sampled and corresponding to amounts of 100, 200, 500, 1000, 1500, and 2000 randomly sampled training datasets are plotted according to each model performance metric. Test sets reporting results are identical across models for each outcome variable. LE: law enforcement.

**Figure 3.**
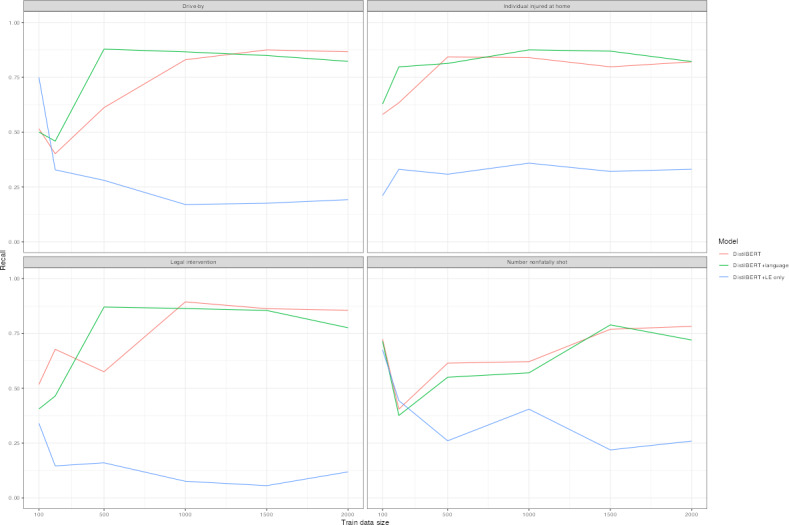
Learning curve by outcome, model type—recall. Recall scores are plotted for distilBERT models, distilBERT models with language replacement, and models that do not use LE narratives. Training data randomly sampled and corresponding to amounts of 100, 200, 500, 1000, 1500, and 2000 randomly sampled training datasets are plotted according to each model performance metric. Test sets reporting results are identical across models for each outcome variable. LE: law enforcement.

**Figure 4.**
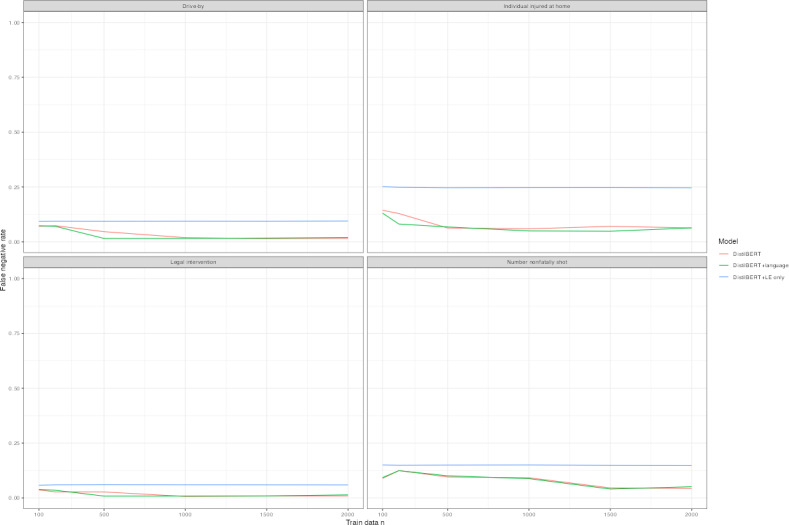
Learning curve by outcome, model type—false negative rate. False negative scores are plotted for distilBERT models, distilBERT models with language replacement, and models that do not use LE narratives. Training data randomly sampled and corresponding to amounts of 100, 200, 500, 1000, 1500, and 2000 randomly sampled training datasets are plotted according to each model performance metric. Test sets reporting results are identical across models for each outcome variable. LE: law enforcement.

Table 3 displays classification performance by *F*_1_-score for each model type. Training data of approximately 1500 cases achieved an *F*_1_-score of at least 0.6 for each outcome, though at 1000 cases, the majority of outcomes was at or exceeding 0.6. The exception was the number nonfatally shot. Figure 1 plots learning curves by *F*_1_-score in Table 3. Replacement language models tended to perform best (Table 3 and Figure 1) with the highest *F*_1_-score in all save 6 model interactions. In particular, language replacement models consistently obtained the highest *F*_1_-score for legal intervention homicides (Table 3 and Figure 1). Similarly, language replacement models featured higher precision scores for a subset of outcomes (Figure 2 and Table S4 in Multimedia Appendix 1). Less substantial difference occurred with recall (Figure 3 and Table S4 in Multimedia Appendix 1) and the false negative rate (Figure 4) between models. Omitting coroner or medical examiner reports performed worse across outcomes (Figures 1-4). Language replacement models trained on 1500‐2000 narratives obtained low false negative rates ranging from 1% to 5% of true cases resulting in a misclassified outcome (Figure 1 and Table S4 in Multimedia Appendix 1).

**Table 3.** *F*_1_-scores by model outcome, training data, and model type.

Outcome	Train, n	DistilBERT^a^, *F*_1_-score	DistilBERT+LE^b^ only^c^, *F*_1_-score	DistilBERT+language^d^, *F*_1_-score
Drive-by	100	*0.219^e^*	0.168	0.209
Drive-by	200	*0.232*	0.148	0.232
Drive-by	500	0.381	0.144	*0.473*
Drive-by	1000	*0.626*	0.124	0.606
Drive-by	1500	0.619	0.126	*0.623*
Drive-by	2000	0.593	0.126	*0.635*
Police shooting	100	0.231	0.105	*0.305*
Police shooting	200	0.218	0.083	*0.364*
Police shooting	500	0.490	0.083	*0.653*
Police shooting	1000	0.739	0.064	*0.795*
Police shooting	1500	0.771	0.056	*0.856*
Police shooting	2000	0.770	0.080	*0.833*
Number nonfatally shot	100	*0.319*	0.246	0.312
Number nonfatally shot	200	0.281	0.226	*0.286*
Number nonfatally shot	500	0.341	0.192	*0.345*
Number nonfatally shot	1000	0.352	0.220	*0.413*
Number nonfatally shot	1500	0.591	0.182	*0.642*
Number nonfatally shot	2000	0.608	0.195	*0.663*
Individual injured at home	100	0.547	0.222	*0.574*
Individual injured at home	200	0.578	0.283	*0.629*
Individual injured at home	500	0.665	0.277	*0.714*
Individual injured at home	1000	*0.722*	0.294	0.697
Individual injured at home	1500	0.737	0.280	*0.749*
Individual injured at home	2000	*0.744*	0.286	0.739

a The base distilBERT model.

b LE: law enforcement.

c The distilBERT model trained only on LE narratives.

d The distilBERT model where text replacement for uncommon language in the narratives is replaced for clarify.

e Values in italics format correspond to the best *F*_1_-score across the listed models.

**Figure 5.**
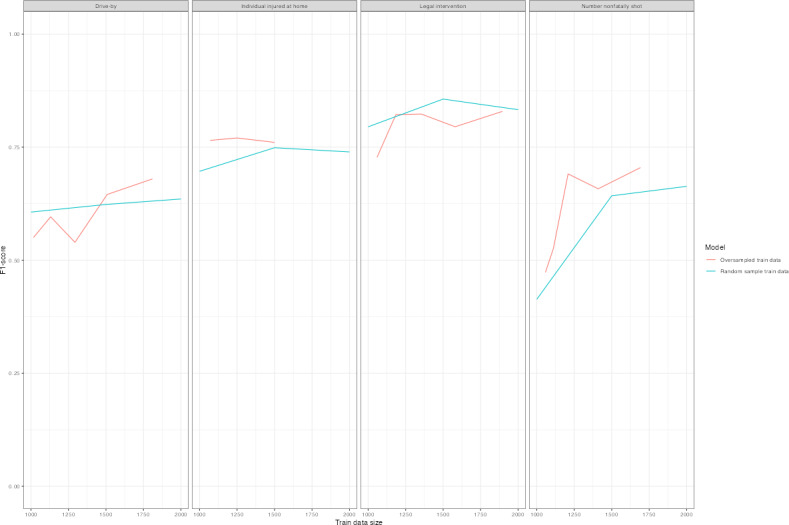
*F*_1_-learning curve for oversampled positive class cases versus baseline language replacement model. *F*_1_-scores are plotted for distilBERT models fit with language replacement for both randomly sampled training data and oversampled training data. Oversampled training data correspond to an increment of a 10% increase in the proportion of positive class cases included in training data. The exact training dataset counts are in Table S4 in Multimedia Appendix 1. Random train data is plotted at 1000, 1500, and 2000 randomly sampled training data records for reference.

Oversampling positive class cases was negligibly helpful in improving *F*_1_-scores (Figure 5). For instance, oversampling for legal intervention homicide to be composed of 20% positive class cases resulted in the addition of 580 positive class cases added to training data and an *F*_1_-score of 0.795 (Table S5 in Multimedia Appendix 1 and Figure 5). Relative to adding 500 randomly sampled cases, which would result in an *F*_1_-score of 0.771 (Table S5 in Multimedia Appendix 1), the *F*_1_-score gain from oversampling was 0.024 (0.795-0.771) and therefore modest.

**Figure 6.**
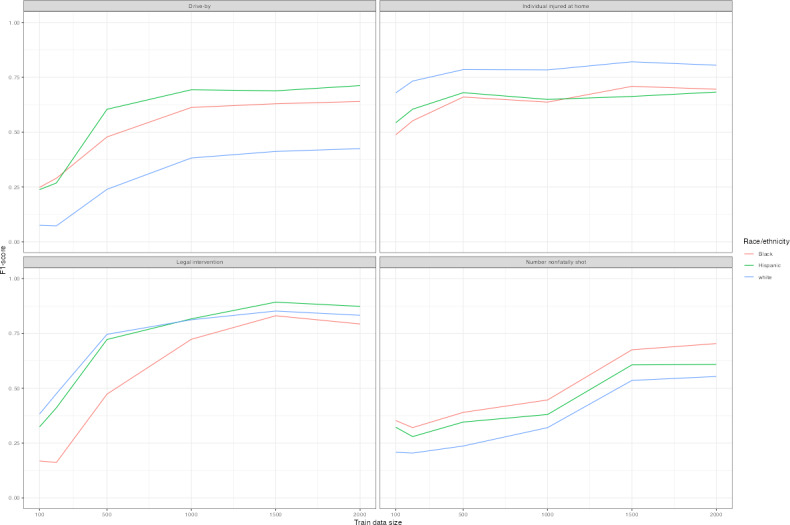
*F*_1_-learning curves for distilBERT+language models by race and ethnicity. *F*_1_-scores are plotted for distilBERT models with language replacement for each outcome by race or ethnicity. Training data randomly sampled and corresponding to amounts of 100, 200, 500, 1000, 1500, and 2000. Test sets reporting results are identical across models for each outcome variable.

**Figure 7.**
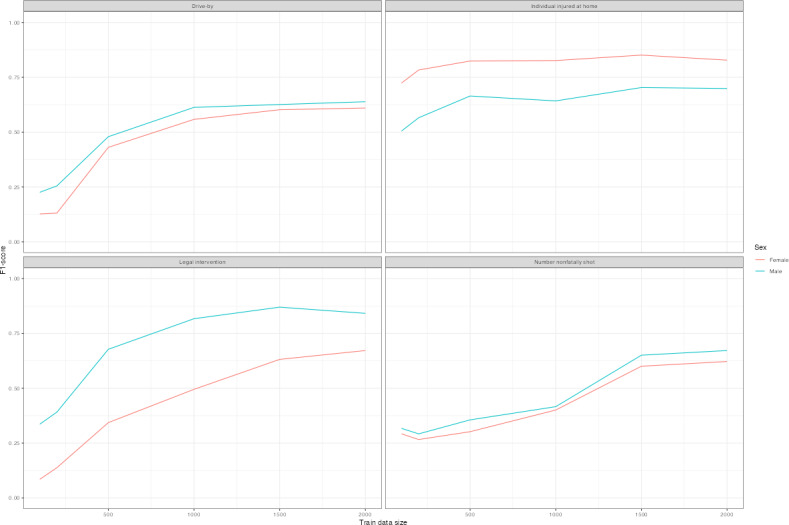
*F*_1_-learning curves for distilBERT+language models by sex. *F*_1_-scores are plotted for distilBERT models with language replacement for each outcome by sex. Training data randomly sampled and corresponding to amounts of 100, 200, 500, 1000, 1500, and 2000. Test sets reporting results are identical across models for each outcome variable.

Figure 6 plots *F*_1_-scores of distilBERT language replacement models, as these models tended to perform best overall and may capture linguistic differences most accurately across subgroups. Predictions differ by race or ethnicity and sex across models. Legal intervention homicide victims who were White or Hispanic were most often correctly classified as such, and Black decedents were least likely to be correctly classified (Figure 6 and Table 4). The prediction difference is substantial for legal intervention victims with lower amounts of training data, though the gap persisted with higher volumes of training data. White decedents shot at home were most often correctly predicted, while Black and Hispanic decedents were least likely. Female decedents were less often correctly predicted than male decedents except if they were shot at home (Figure 7). Among models with at least 1500 records of training data, *F*_1_-score disparities ranged from 0.2 to 0.25 by race and ethnicity, and between male and female decedents with differences ranging from 0.12 to 0.2 (Table 4).

**Table 4.** Classification performance for language replacement models by outcome by subgroup^a^.

Category	Train^b^, n	White, *F*_1_-score	Black, *F*_1_-score	Hispanic, *F*_1_-score	Male, *F*_1_-score	Female, *F*_1_-score
Drive-by	100	0.208	0.353	0.322	0.317	0.292
Drive-by	200	0.204	0.321	0.280	0.292	0.266
Drive-by	500	0.237	0.390	0.346	0.356	0.302
Drive-by	1000	0.321	0.447	0.380	0.416	0.401
Drive-by	1500	0.536	0.675	0.607	0.651	0.600
Drive-by	2000	0.554	0.704	0.608	0.672	0.622
Legal intervention	100	0.383	0.169	0.324	0.336	0.085
Legal intervention	200	0.475	0.162	0.411	0.391	0.138
Legal intervention	500	0.746	0.473	0.722	0.678	0.343
Legal intervention	1000	0.812	0.723	0.816	0.817	0.495
Legal intervention	1500	0.852	0.830	0.893	0.870	0.632
Legal intervention	2000	0.833	0.793	0.873	0.842	0.672
Number nonfatally shot	100	0.075	0.248	0.238	0.226	0.127
Number nonfatally shot	200	0.073	0.290	0.268	0.255	0.131
Number nonfatally shot	500	0.239	0.478	0.604	0.479	0.431
Number nonfatally shot	1000	0.382	0.613	0.693	0.613	0.558
Number nonfatally shot	1500	0.412	0.629	0.688	0.626	0.602
Number nonfatally shot	2000	0.425	0.640	0.712	0.639	0.610
Individual injured at home	100	0.679	0.488	0.543	0.505	0.724
Individual injured at home	200	0.733	0.552	0.605	0.566	0.783
Individual injured at home	500	0.785	0.660	0.680	0.665	0.824
Individual injured at home	1000	0.784	0.637	0.649	0.642	0.826
Individual injured at home	1500	0.821	0.709	0.663	0.703	0.851
Individual injured at home	2000	0.805	0.696	0.683	0.699	0.828

a *F*_1_-scores are listed for distilBERT models with language replacement across target outcomes within subgroups including race, ethnicity, and sex. Test sets reporting results are identical across models for each outcome variable.

b Training data randomly sampled and corresponding to amounts of 100, 200, 500, 1000, 1500, and 2000.

## Discussion

### Principal Findings

This analysis simulated the NLP model-fitting process to demonstrate how different training and preprocessing decisions impact model performance in the supervised classification of violent death narratives. Results show that the compact LLM approach is useful for predicting rare NVDRS outcomes relative to naive prediction baselines. The best model for drive-by shootings achieved an *F*_1_-score of 0.635 (Table 3) for an outcome, where the proportion of positive class cases was 9.2%. For context, if the model had correctly classified only the 9.2% of positive class cases, it would have achieved an *F*_1_-score of 0.162. While variation exists across outcomes in the rate of improvement over a naive prediction, the improvement in *F*_1_-scores across infrequent NVDRS events demonstrates that a compact LLM approach is useful.

Simulations suggest that fine-tuning compact LLMs on NVDRS text requires approximately 1000‐1500 training data records to achieve an *F*_1_-score of at least 0.6. However, substantial variation existed between outcomes. For drive-by shootings and for whether a victim is injured at home, the learning curves flatten at 1000 cases and do not make further *F*_1_-score gains with the addition of additional training data. Legal intervention (police shootings) continues to make additional *F*_1_-score gains beyond 1000 cases and achieve an *F*_1_-score of 0.75 at 2000 cases. Similarly, for the number of victims nonfatally shot, the addition of training data beyond 1000 cases substantially improves the *F*_1_-score to 0.66 at 2000 cases.

In addition, preprocessing data to reduce domain-specific jargon resulted in improved model performance. Oversampling the positive class cases in training data does not increase prediction accuracy substantially over randomly sampled training data. Predictions differed by race, ethnicity, and sex.

Results suggested that compact LLMs are useful but require training data to correctly classify outcomes of interest. Random sampling and labeling a sufficient number of cases (approximately 1000) combined with a weighting layer is an effective classification strategy. Relative to recent few-shot and zero-shot learning applications using similar data sources [29], simulation findings differ, in that the volume of training data required is more substantial. The additional training data may be a function of a class imbalance in the target outcome, as other applications use more prevalent outcomes.

Differential prediction by subgroup is not explainable by outcome frequency or narrative length alone. For instance, White decedents of police shootings are less prevalent than Black decedents in the sample but are more often classified correctly. Similarly, female decedents have longer median narratives for all outcomes but are less likely to be correctly classified. This finding expands upon the current literature, which has found systematic data missingness in NVDRS [28,38-40]. Further research should characterize sources of differential prediction, whether input narratives or exacerbation by NLP classifier, and examine fairness-aware models particularly if the prediction is used for decision-making or resource allocation in public health settings.

### Limitations

This research is subject to several limitations. First, results from a compact LLM may not fully generalize to new LLMs with additional sophistication or to different language contexts beyond NVDRS. Label noise from NVDRS annotators may mean that results understate the performance of compact LLMs, which is consistent with police shootings tending to be the outcome type that is most accurately predicted. The potential for differential prediction by subgroup raises concerns about fairness and equity in model performance. Further investigations into the sources of this differential prediction are needed to ensure that NLP applications do not exacerbate existing disparities.

### Conclusions

Compact LLMs with simple text changes can effectively predict rare NVDRS outcomes. For researchers using supervised machine learning to expand knowledge of violent deaths beyond existing coded fields, applying compact LLMs to sufficient training data can be a valuable approach. While future advancements will likely improve access to privacy-compliant, more sophisticated LLMs for analyzing sensitive data, this study provides a useful baseline for researchers pursuing similar efforts in the interim while underlining the potential for differential prediction by subgroup.

## Supplementary material

10.2196/68212 Multimedia Appendix 1 Additional model performance metrics.

## Abbreviations

*ICD*: *International Classification of Diseases*
LE: law enforcement
LLM: large language model
NLP: natural language processing
NVDRS: National Violent Death Reporting System
