# Supplementary material for: Supervised Natural Language Processing Classification of Violent Death Narratives: Development and Assessment of a Compact Large Language Model
Source: JMIR AI. 2025 Jun 19;4:e68212. doi: 10.2196/68212 (PMC12223685; doi:10.2196/68212)
Supplement: Multimedia Appendix 1 [file ai-v4-e68212-s001.docx]

**Table S1.** Language replacement words and replacement value.

| **Replaced Words** | **Value** |
| --- | --- |
| Gsw, gsws, gun shot wound | Gunshot wound |
| Vic, V, V’s, Vic’s | Victim |
| S, S’s | Suspect |
| Law enforcement, LE, LEO, officer, police officer, officers, cop, cops, sheriff, sheriffs, detective, trooper, troopers, marshall, marshals , deputy, deputies | Police |

**Table S2.** Model parameters.

| **Parameter** | **Value** |
| --- | --- |
| Learning rate | 0.0005 |
| Batch size | 60 |
| Number of epochs | 5 |
| Weight decay | 0.01 |

**Table S3.** Summary of simulations.

| **Simulation** | **Type** | **Training Data** | **Training Data N** |
| --- | --- | --- | --- |
| 1 - distilBERT | Model fitting | Random sample | 100, 200, 500, 1000, 1500, 2000 |
| 2 - distilBERT + oversample | Pre-processing | Oversampled positive class outcome | 1000, 10%, 20%, 30% , 40%, 50% |
| 3 - Distilbert + Language | Pre-processing | Random Sample; Language replacement | 100, 200, 500, 1000, 1500, 2000 |
| 4 - distilBERT + LE only | Pre-processing | Random Sample; Law enforcement narrative only | 100, 200, 500, 1000, 1500, 2000 |

**Table S4.** Classification performance by oversampled proportion of positive class case.

| **Outcome** | **Train Proportion** | **Train N** | **F1** | **Precision** | **Recall** |
| --- | --- | --- | --- | --- | --- |
| Driveby | Random Sample | 1000 | 0.626 | 0.503 | 0.830 |
| Driveby | 10 percent | 1016 | 0.550 | 0.419 | 0.802 |
| Driveby | 20 percent | 1131 | 0.596 | 0.451 | 0.877 |
| Driveby | 30 percent | 1293 | 0.539 | 0.383 | 0.913 |
| Driveby | 40 percent | 1508 | 0.645 | 0.513 | 0.869 |
| Driveby | 50 percent | 1810 | **0.680** | 0.598 | 0.788 |
| Police Shooting | Random Sample | 1000 | 0.739 | 0.630 | 0.894 |
| Police Shooting | 10 percent | 1059 | 0.727 | 0.633 | 0.854 |
| Police Shooting | 20 percent | 1185 | 0.822 | 0.745 | 0.916 |
| Police Shooting | 30 percent | 1354 | 0.823 | 0.741 | 0.926 |
| Police Shooting | 40 percent | 1580 | 0.795 | 0.682 | 0.954 |
| Police Shooting | 50 percent | 1896 | **0.829** | 0.734 | 0.953 |
| Number Nonfatally Shot | Random Sample | 1000 | 0.352 | 0.245 | 0.621 |
| Number Nonfatally Shot | 10 percent | 1111 | 0.526 | 0.506 | 0.547 |
| Number Nonfatally Shot | 20 percent | 1058 | 0.473 | 0.357 | 0.701 |
| Number Nonfatally Shot | 30 percent | 1209 | 0.691 | 0.616 | 0.786 |
| Number Nonfatally Shot | 40 percent | 1410 | 0.658 | 0.530 | 0.868 |
| Number Nonfatally Shot | 50 percent | 1692 | **0.705** | 0.597 | 0.860 |
| Victim Injured at Home | Random Sample | 1000 | 0.722 | 0.633 | 0.840 |
| Victim Injured at Home | 30 percent | 1071 | **0.765** | 0.734 | 0.799 |
| Victim Injured at Home | 40 percent | 1250 | 0.770 | 0.747 | 0.795 |
| Victim Injured at Home | 50 percent | 1500 | 0.761 | 0.679 | 0.864 |

Notes: Each numeric cell contains performance (F1, precision, recall) for each model outcome by the proportion of positive class cases that were included in the training data. In Column 2, the proportion of the training data that was a positive class case is listed, with the exception being “random sample” which denotes training data that is randomly sampled up to n=1000 cases and is presented for reference. The train proportion increases the proportion of positive class cases beyond the random sample. The exception is the outcome Victim Injured at Home which omits the train proportion at 10 and 20 percent because the randomly sampled proportion exceeds 20 percent.

**Table S5.** Classification metrics for language replacement models.

| **Category** | **Train N** | **F1** | **Precision** | **Recall** | **FNR** |
| --- | --- | --- | --- | --- | --- |
| Driveby | 100 | 0.21 | 0.13 | 0.5 | 0.07 |
| Driveby | 200 | 0.23 | 0.15 | 0.46 | 0.07 |
| Driveby | 500 | 0.47 | 0.32 | 0.88 | 0.02 |
| Driveby | 1000 | 0.61 | 0.47 | 0.87 | 0.02 |
| Driveby | 1500 | 0.62 | 0.49 | 0.85 | 0.02 |
| Driveby | 2000 | 0.64 | 0.52 | 0.82 | 0.02 |
| Legal Intervention | 100 | 0.3 | 0.24 | 0.41 | 0.04 |
| Legal Intervention | 200 | 0.36 | 0.3 | 0.46 | 0.03 |
| Legal Intervention | 500 | 0.65 | 0.52 | 0.87 | 0.01 |
| Legal Intervention | 1000 | 0.79 | 0.74 | 0.86 | 0.01 |
| Legal Intervention | 1500 | 0.86 | 0.86 | 0.85 | 0.01 |
| Legal Intervention | 2000 | 0.83 | 0.9 | 0.78 | 0.01 |
| Number Nonfatally Shot | 100 | 0.31 | 0.2 | 0.71 | 0.09 |
| Number Nonfatally Shot | 200 | 0.29 | 0.23 | 0.38 | 0.12 |
| Number Nonfatally Shot | 500 | 0.34 | 0.25 | 0.55 | 0.1 |
| Number Nonfatally Shot | 1000 | 0.41 | 0.32 | 0.57 | 0.09 |
| Number Nonfatally Shot | 1500 | 0.64 | 0.54 | 0.79 | 0.04 |
| Number Nonfatally Shot | 2000 | 0.66 | 0.62 | 0.72 | 0.05 |
| Victim Injured at Home | 100 | 0.57 | 0.53 | 0.63 | 0.13 |
| Victim Injured at Home | 200 | 0.63 | 0.52 | 0.8 | 0.08 |
| Victim Injured at Home | 500 | 0.71 | 0.64 | 0.81 | 0.07 |
| Victim Injured at Home | 1000 | 0.7 | 0.58 | 0.88 | 0.05 |
| Victim Injured at Home | 1500 | 0.75 | 0.66 | 0.87 | 0.05 |
| Victim Injured at Home | 2000 | 0.74 | 0.67 | 0.82 | 0.06 |

Notes: Each numeric cell contains performance (F1, precision, recall) for each model outcome by the proportion of positive class cases that were included in the training data. In Column 2, the proportion of the training data that was a positive class case is listed, with the exception being “random sample” which denotes training data that is randomly sampled up to n=1000 cases and is presented for reference. The train proportion increases the proportion of positive class cases beyond the random sample. The exception is the outcome Victim Injured at Home which omits the train proportion at 10 and 20 percent because the randomly sampled proportion exceeds 20 percent.
